# Progressive scattered and reticular pigmentation lesions

**DOI:** 10.1016/j.jdcr.2025.08.016

**Published:** 2025-08-29

**Authors:** Yao Zhou, Lining Huang, Chao Yang, Zhijun Xu, Amira Elbendary, Ruzeng Xue

**Affiliations:** a Department of Dermatology, Dermatology Hospital, Southern Medical University, Guangzhou, China; b Department of Dermatology, Kasr Alainy Faculty of Medicine, Cairo University, Giza, Egypt

**Keywords:** Dowling-Degos disease, genodermatosis, hyperpigmentation, KRT5 mutation, melanosome

## Clinical case

**Fig 1:**
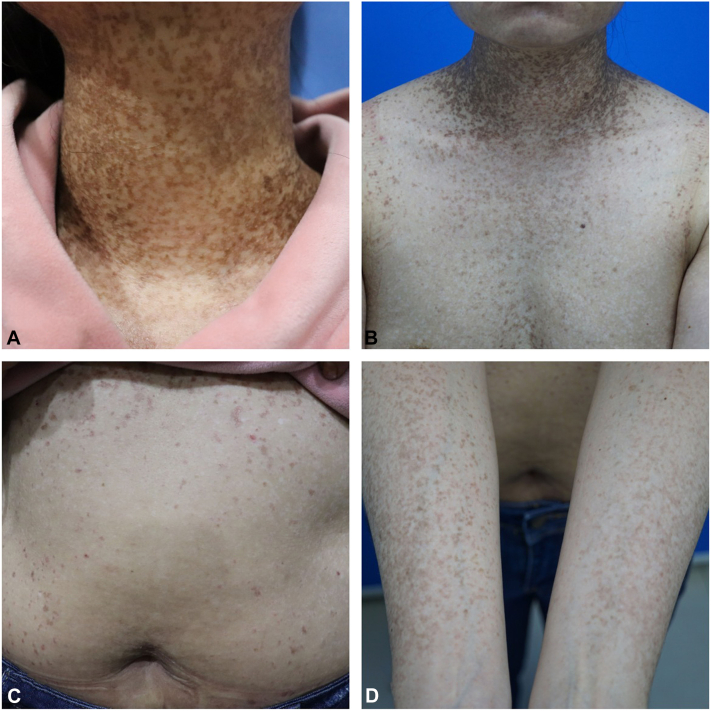
Clinical manifestation of the patient **(A-D)**.

**Fig 2:**
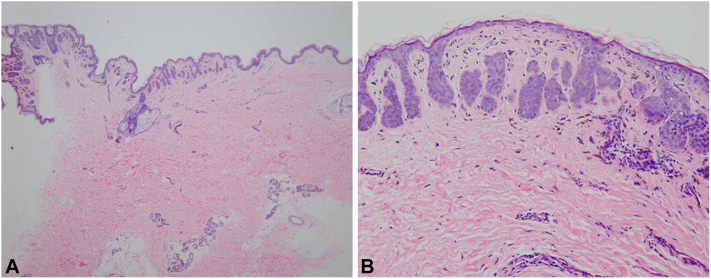
Skin biopsy of the patient **(A, B)**. Elongated rete ridges, scattered phagocytic melanophages in the shallow dermis, and a few lymphocytes around vessels.

**Fig 3:**
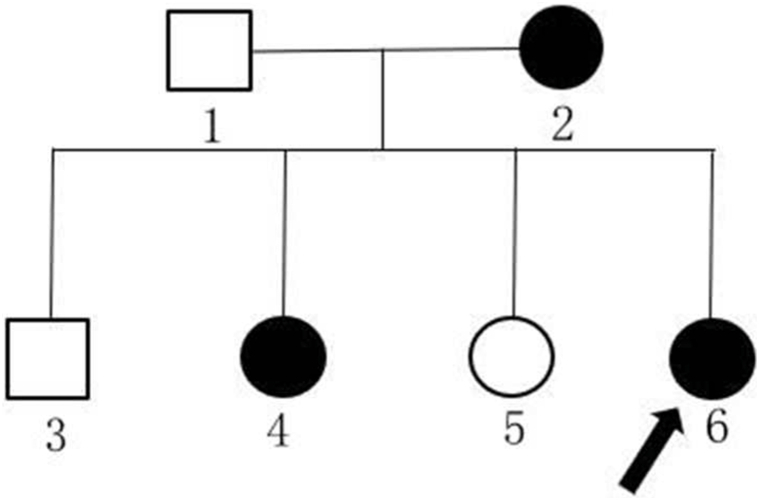
Pedigree of the family. The proband (individual 6) shares a similar phenotype with her mother (individual 2) and elder sister (individual 4).

A 40-year-old woman presented with a 25-year history of scattered hyperpigmented round macules on the face and reticulated hyperpigmentation and reddish-brown keratotic papules on the neck, axillae, inframammary areas, groin, and forearms (Fig 1, *A-D*). Pruritus was triggered by hot weather and sun exposure. Histopathology showed increased pigment granules of the basal layer, elongated finger-like rete ridges, scattered melanophages in the papillary dermis, and a sparse perivascular lymphocytic infiltrate (Fig 2, *A*, *B*). Besides, her mother and one of her elder sisters also had similar clinical conditions (Fig 3). DNA sequencing from the family revealed a heterozygous 2-base pair deletion at positions 442 to 443 in exon 1 of the *KRT5* gene. Systemic etretinate and 0.1% topical tretinoin cream showed no success in the patients.

**Question 1: What is the most likely diagnosis?**

**A.** Dowling-Degos disease (DDD) (reticulate pigmented anomaly of the flexures)
**B.** Reticulate acropigmentation of Kitamura (RAPK)
**C.** Haber syndrome
**D.** Neurofibromatosis type 1 (NF1)
**E.** Acanthosis nigricans

**Answers**

**A.** DDD – Correct. DDD is a rare hereditary progressive pigmentation disorder. Affected individuals usually present with symptoms in the third to fourth decade of life.^1^ It is characterized by scattered and reticulate hyperpigmentation of the flexures, which usually appears on the axillae, large skinfolds, trunk, and extremities. It shows filiform or antler-like elongation of rete ridges of the epidermis and hyperpigmentation in the lower third of the elongated rete ridges histologically.^1^ The main genes implicated in DDD pathogenesis are *KRT5*, *POGLUT1*, *POFUT1*, and *PSENEN.* Flexural DDD was initially found to be caused by loss of mutations affecting the *KRT5* gene region.^2^
**B.** RAPK – Incorrect. RAPK usually presents in the first or second decade of life, manifesting as hyperpigmented macules on the dorsal aspects of the hands and feet and gradually extending proximally. It shows epidermal atrophy, elongation of the rete ridges, and hyperpigmentation at the basal layer histologically.^1^ The mutation in *ADAM10* encodes a zinc metalloprotease in the RAPK family. It is reported that increased E-cadherin proteolysis and blister formation in eczematous dermatitis is correlated with increased *ADAM10* expression positively.^3^
**C.** Haber syndrome – Incorrect. Haber syndrome usually has its onset in adolescence. It is characterized by initial photosensitive rosacea-like facial erythema, multiple keratotic papules, pitted scars, comedone-like lesions, and reticulate hyperpigmentation. Rashes usually appear on the trunk, axillae, and proximal extremities. It shows follicular keratotic plugs epidermal budding histologically.^1^
**D.** NF1 – Incorrect. NF1 is inherited in an autosomal dominant pattern, and a single copy of a mutated or deleted *NF1* gene is needed in order to be effective. Intertriginous freckling is 1 of the 7 cardinal diagnostic features of NF1. It usually shows in the axillae and inguinal folds.^4^
**E.** Acanthosis nigricans – Incorrect. Acanthosis nigricans is associated with obesity, insulin resistance, diabetes mellitus, endocrine disorders, and internal malignancy. It is characterized by thick, velvety, brownish-black, hyperkeratotic plaques. The main sites of involvement are the intertriginous and flexural areas. It shows exophytic papillomatous epidermal proliferations in church spire patterns and no increase in pigment histologically.^5^

## Conflicts of interest

None disclosed.
